# Survival of soft tissue sarcoma patients after completing six cycles of first-line anthracycline containing treatment: an EORTC-STBSG database study

**DOI:** 10.1186/s13569-020-00137-5

**Published:** 2020-09-09

**Authors:** Arie Jan Verschoor, Saskia Litière, Sandrine Marréaud, Ian Judson, Maud Toulmonde, Eva Wardelmann, Axel LeCesne, Hans Gelderblom

**Affiliations:** 1 Department of Medical Oncology, Leiden University Medical Centre, Albinusdreef 2, 2333 ZA Leiden, The Netherlands; 2 European Organisation for Research and Treatment of Cancer, Avenue Emmanuel Mounier 83/11, 1200 Brussels, Belgium; 3 Institute of Cancer Research and the Royal Marsden NHS Foundation Trust, 123 Old Brompton Road, London, SW7 3RP UK; 4 Institut Bergonié, 229 Cours de l’Argonne, 33000 Bordeaux, France; 5 Gerhard Domagk Institute of Pathology, University Hospital Muenster, Domagkstraße 17, 48149 Muenster, Germany; 6 Institut Gustave Roussy, 114 Rue Edouard Vaillant, 94800 Villejuif, France; 7 Department of Medical Oncology, Leiden University Medical Center, P.O. box 9600, 2300 RC Leiden, The Netherlands

**Keywords:** Soft tissue sarcoma, Doxorubicin, Prognosis, Ifosfamide, Overall survival, Progression-free survival

## Abstract

**Background:**

Doxorubicin based chemotherapy is standard first line treatment for patients with soft tissue sarcoma. Currently several options to improve survival after doxorubicin based chemotherapy are being studied. This study reports on survival after completing 6 cycles of doxorubicin containing first line treatment, which is important when designing studies trying to improve outcomes of first line treatment.

**Methods:**

A retrospective database analysis was performed on 2045 patients from 12 EORTC sarcoma trials (inclusion period 1980–2012) receiving first line doxorubicin based chemotherapy for advanced soft tissue sarcoma in order to establish progression free survival and overall survival after completing 6 cycles of first line doxorubicin based chemotherapy. Endpoints were overall survival and progression free survival. Factors studied were histologic subtype and type of doxorubicin chemotherapy.

**Results:**

748 of 2045 (36.6%) received at least 6 cycles and did not progress during or at the end of chemotherapy. 475 of 2045 (23.2%) of patients received exactly 6 cycles and did not progress during or at the end of chemotherapy. Median progression free survival after 6 cycles of doxorubicin based chemotherapy was 4.2 months (95% confidence interval 3.7–4.8) and median overall survival 15.7 months (14.0–17.8). Median progression free survival and overall survival from randomisation/registration were 8.7 months (95% confidence interval 8.2–9.1) and 20.1 months (95% confidence interval 18.3–22.3) respectively. Significant differences in progression free survival were found between chemotherapy regimens, but not for overall survival. These data are also reported for patients receiving 7 or more cycles of chemotherapy and for patients with 3 or more cycles of chemotherapy.

**Conclusion:**

This large retrospective study is the first to report progression free survival and overall survival after completion of 6 cycles of first line doxorubicin containing chemotherapy. These results are important when designing new studies exploring for example maintenance therapy after doxorubicin based chemotherapy.

## Background

Soft tissue sarcomas (STS) are a rare group of tumours comprising approximately 1% of all cancers and containing approximately 70 different histological entities [1]. Clinical behaviour differs between the various histological entities [1]. Surgery is the primary treatment for localized disease when resection is possible with the option of adding neo-adjuvant or adjuvant radiotherapy [2]. For patients with locally advanced and/or distant metastatic disease the goal of treatment is to prolong survival and treatment mainly consists of systemic treatment, e.g. cytotoxic drugs and tyrosine kinase inhibitors [2].

The current first line chemotherapy consists of anthracycline based chemotherapy either as monotherapy or combination therapy [3]. Survival remains poor for patients presenting with incurable disease. Overall survival (OS) with doxorubicin monotherapy is approximately 12.8 months and with doxorubicin/ifosfamide combination therapy approximately 14.3 months [3]. More recent trials report slightly better median OS for doxorubicin monotherapy with 17.6 months (GeDDiS), 16.9 months (PICASSO III) and 19.0 months (SARC021) [4–6]. Although the phase II results of the addition of olaratumab to doxorubicin were promising and resulted in a temporary approval by both the U.S. Food and Drug Administration and the European Medical Agency, the results of the phase III ANNOUNCE trial were negative and the registration of olaratumab was withdrawn [7, 8]. These phase III results did not show any difference in overall survival for patients treated with the addition of olaratumab to doxorubicin compared to patients with doxorubicin with placebo (median overall survival 20.4 months (with olaratumab) vs. 19.7 months without) [8].

Now, other treatment strategies have to be studied to increase the PFS and OS of STS patients including the addition of maintenance therapy after completing six cycles of doxorubicin. In order to assist in the design of maintenance studies it is important to have survival data of patients after completing six cycles of doxorubicin containing treatment and to understand the extent of the attrition in the number of patients available for study, indeed the percentage who could possibly benefit from maintenance therapy by not having progressed before completing 6 cycles of treatment. This study reports the OS data of study patients completing six cycles of anthracycline or anthracycline combination therapy in the European Organisation for Research and Treatment of Cancer Soft Tissue and Bone Sarcoma Group trial database.

## Methods

### Patients

The European Organisation for Research and Treatment of Cancer Soft Tissue and Bone Sarcoma Group study database contains data from 12 trials studying doxorubicin alone or in combination with ifosfamide (patients were included in the different studies between 1980 and 2012) [3, 9–19]. All but one study, included patients with locally advanced or metastatic STS. The study by Steward et al*.* only included patients with metastatic STS [12]. Patients with at least 1 cycle of treatment were considered for this study. Reasons for exclusion were previous treatment with chemotherapy either as adjuvant or palliative treatment, patients without data on progression and death and patients diagnosed with Gastrointestinal Stromal Tumour (GIST). Among these patients, we focused on patients who did not progress before the end of treatment. End of treatment was considered to be 21 days after the date of administration of the last treatment (Additional file 1: Figure S1). Analysis was done in three different subgroups: patients who received exactly 6 cycles of doxorubicin containing chemotherapy, patients with 7 or more cycles and patients with less than 6 cycles who stopped treatment for reasons other than progression.

The EORTC studies 62012, 62061, 62091, 62962 and 62971 had treatment regimens including a maximum number of 6 cycles of doxorubicin, 62941 7 cycles and the other studies aimed for a cumulative dose of 550 mg/m^2^ of doxorubicin allowing for more if the ejection fraction remained within certain limits.

### Endpoints

Endpoints were PFS and OS after completing treatment, because the aim of the study was to determine PFS and OS after completion of 6 cycles of doxorubicin containing treatment in patients who did not have progressive disease at that time point. PFS was defined as the time between end of treatment and progression or death. OS was defined as the time between end of treatment and death. Also calculated were PFS from date of randomisation to date of progression or death and OS from date of randomisation to date of death. Patients progressing between start of treatment and 21 days after the last administration date were not considered for the PFS and OS after treatment analysis, because only those patients who do not have progression before the start of maintenance treatment will qualify for maintenance treatment. Time on treatment was calculated from date of randomisation or registration and the end of treatment.

### Covariates

Patients were grouped according to treatment *i.e.* doxorubicin 75 mg/m^2^ monotherapy, doxorubicin 50 mg/m^2^ combined with ifosfamide 5 g/m^2^, doxorubicin 75 mg/m^2^ combined with ifosfamide 5 g/m^2^ and doxorubicin 75 mg/m^2^ combined with ifosfamide 10 g/m^2^. The other covariate considered in this study was histologic subtype. If central pathology review was available the central pathology diagnosis was used, if it was not present the local pathology diagnosis was used. Only histologic subtypes comprising more than ten percent of patients were considered for separate analysis.

### Statistics

PFS and OS were calculated using the Kaplan Meier method. PFS and OS were compared using a cox proportional hazard model. Significance was set at p = 0.05.

## Results

In total, 2045 patients were included in this study [PFS from randomisation for the complete population was 4.8 months (95% confidence interval 4.4–5.1) and OS from randomisation was 12.4 months (11.9–12.9)]. Almost 50% of patients were treated with doxorubicin 75 mg/m^2^ as monotherapy; the other patients were treated with one of the combination regimens (Additional file 1: Table S1 shows the distribution of patients according to study and treatment regimen. Additional file 1: Table S2 shows the number of treatment cycles by study). Median time on treatment was 15 weeks, corresponding to a median number of 5 cycles. Of all patients, 43.7% of patients (894) were treated with 6 or more cycles of chemotherapy, 70.2% of patients were treated with 3 or more cycles. Five hundred fifty five patients (27.1%) received exactly 6 cycles of chemotherapy. Median follow-up for all patients was 4.1 years [Inter quartile range (IQR) 2.5–6.5 years]. Most of the patients receiving more than 6 cycles, were included in studies studying the doxorubicin 50 mg/m^2^/ifosfamide 5 g/m^2^ regimen (Additional file 1: Table S1).

Of these patients with at least 6 cycles of treatment 748 patients (83.7% of all patients treated with 6 or more cycles) did not progress before or at the end of treatment. For exactly 6 cycles, 475 patients (85.6% of patients treated with exactly 6 cycles) did not progress before the end of treatment. Table 1 shows the percentage of patients considered for this study per treatment strategy.

**Table 1:** Distribution of patients per treatment strategy and number of cycles

	Treatment				
	DOX 75 (N = 948)	DOX 50—IFO 5 (N = 614)	DOX 75—IFO 5 (N = 266)	DOX 75—IFO 10 (N = 217)	Total (N = 2045)
Number of patients with *at least* 6 cycles	403 (42.5)	270 (44.0)	103 (38.7)	118 (54.4)	895 (43.7)
Progression before/at end of treatment	67 (16.6)	55 (20.4)	15 (14.6)	9 (7.6	146 (16.3)
No progression before/at end of treatment	336 (83.4)	215 (79.6)	88 (85.4)	109 (92.4)	748 (83.6)
Number of patients with *less than* 6 cycles	545 (57.4)	344 (56.0)	163 (61.3)	99 (45.6)	1151 (56.3)
Progression before/at end of treatment	312 (57.2)	175 (50.9)	52 (31.9)	28 (28.3)	567 (49.3)
No progression before/at end of treatment	233 (42.8)	168 (49.1)	111 (68.1)	71 (71.7)	584 (50.7)

### Baseline characteristics

Tables 2 and 3 and Additional file 1: Table S1a–d show the characteristics of the included patients. No important differences exist between the different groups. The most common histologic subtype was leiomyosarcoma (31%), followed by the no longer existing histologic entity malignant fibrous histiocytoma (MFH) (13%) and synovial sarcoma (10%) (Additional file 1: Table S3). As none of the other subtypes did comprise ten percent of the patients as an entity, these were considered together when histologic subtype was studied (also MFH was added to the miscellaneous group as this entity no longer exists; smaller subgroups would reduce the statistical power).

**Table 2:** Baseline characteristics

	Less than 6 cycles			Exactly 6 cycles			More than 6 cycles			Exactly 6 cycles—no PD				
	PD before end of treatment (N = 567)	No PD before end of treatment (N = 584)	Total (N = 1151)	PD before end of treatment (N = 80)	No PD before end of treatment (N = 475)	Total (N = 555)	PD before end of treatment (N = 66)	No PD before end of treatment (N = 273)	Total (N = 339)	Treatment				
										DOX 75 (N = 223)	DOX 50-IFO 5 (N = 80)	DOX 75-IFO 5 (N = 63)	DOX 75-IFO 10 (N = 109)	Total (N = 475)
	N (%)	N (%)	N (%)	N (%)	N (%)	N (%)	N (%)	N (%)	N (%)	N (%)	N (%)	N (%)	N (%)	N (%)
Gender														
Male	273 (48.1)	284 (48.6)	557 (48.4)	45 (56.3)	226 (47.6)	271 (48.8)	26 (39.4)	139 (50.9)	165 (48.7)	102 (45.7)	34 (42.5)	30 (47.6)	60 (55.0)	226 (47.6)
Female	294 (51.9)	299 (51.2)	593 (51.5)	35 (43.8)	248 (52.2)	283 (51.0)	40 (60.6)	134 (49.1)	174 (51.3)	121 (54.3)	45 (56.3)	33 (52.4)	49 (45.0)	248 (52.2)
Missing	0 (0.0)	1 (0.2)	1 (0.1)	0 (0.0)	1 (0.2)	1 (0.2)	0 (0.0)	0 (0.0)	0 (0.0)	0 (0.0)	1 (1.3)	0 (0.0)	0 (0.0)	1 (0.2)
Age														
< 40 years	122 (21.5)	124 (21.2)	246 (21.4)	26 (32.5)	125 (26.3)	151 (27.2)	18 (27.3)	80 (29.3)	98 (28.9)	45 (20.2)	23 (28.8)	26 (41.3)	31 (28.4)	125 (26.3)
40–50 years	137 (24.2)	122 (20.9)	259 (22.5)	20 (25.0)	115 (24.2)	135 (24.3)	11 (16.7)	64 (23.4)	75 (22.1)	54 (24.2)	15 (18.8)	9 (14.3)	37 (33.9)	115 (24.2)
50–60 years	164 (28.9)	170 (29.1)	334 (29.0)	19 (23.8)	148 (31.2)	167 (30.1)	13 (19.7)	73 (26.7)	86 (25.4)	77 (34.5)	19 (23.8)	14 (22.2)	38 (34.9)	148 (31.2)
> = 60 years	134 (23.6)	156 (26.7)	290 (25.2)	13 (16.3)	85 (17.9)	98 (17.7)	16 (24.2)	49 (17.9)	65 (19.2)	47 (21.1)	21 (26.3)	14 (22.2)	3 (2.8)	85 (17.9)
Missing	10 (1.8)	12 (2.1)	22 (1.9)	2 (2.5)	2 (0.4)	4 (0.7)	8 (12.1)	7 (2.6)	15 (4.4)	0 (0.0)	2 (2.5)	0 (0.0)	0 (0.0)	2 (0.4)
Performance status														
PS 0	223 (39.3)	265 (45.4)	488 (42.4)	38 (47.5)	274 (57.7)	312 (56.2)	25 (37.9)	127 (46.5)	152 (44.8)	132 (59.2)	39 (48.8)	38 (60.3)	65 (59.6)	274 (57.7)
PS 1	275 (48.5)	265 (45.4)	540 (46.9)	34 (42.5)	189 (39.8)	223 (40.2)	32 (48.5)	120 (44.0)	152 (44.8)	84 (37.7)	37 (46.3)	24 (38.1)	44 (40.4)	189 (39.8)
PS 2 +	67 (11.8)	51 (8.7)	118 (10.3)	8 (10.0)	11 (2.3)	19 (3.4)	9 (13.6)	24 (8.8)	33 (9.7)	7 (3.1)	3 (3.8)	1 (1.6)	0 (0.0)	11 (2.3)
Missing	2 (0.4)	3 (0.5)	5 (0.4)	0 (0.0)	1 (0.2)	1 (0.2)	0 (0.0)	2 (0.7)	2 (0.6)	0 (0.0)	1 (1.3)	0 (0.0)	0 (0.0)	1 (0.2)

**Table 3:** Tumour and treatment characteristics

	Less than 6 cycles			Exactly 6 cycles			More than 6 cycles			Exactly 6 cycles—no PD				
	PD before end of treatment (N = 567)	No PD before end of treatment (N = 584)	Total (N = 1151)	PD before end of treatment (N = 80)	No PD before end of treatment (N = 475)	Total (N = 555)	PD before end of treatment (N = 66)	No PD before end of treatment (N = 273)	Total (N = 339)	Treatment				
										DOX 75 (N = 223)	DOX 50-IFO 5 (N = 80)	DOX 75-IFO 5 (N = 63)	DOX 75-IFO 10 (N = 109)	Total (N = 475)
	N (%)	N (%)	N (%)	N (%)	N (%)	N (%)	N (%)	N (%)	N (%)	N (%)	N (%)	N (%)	N (%)	N (%)
Histopathological grading														
Grade I and II	38 (6.7)	30 (5.1)	68 (5.9)	6 (7.5)	52 (10.9)	58 (10.5)	8 (12.1)	24 (8.8)	32 (9.4)	26 (11.7)	12 (15.0)	8 (12.7)	6 (5.5)	52 (10.9)
Grade III	331 (58.4)	366 (62.7)	697 (60.6)	46 (57.5)	331 (69.7)	377 (67.9)	33 (50.0)	152 (55.7)	185 (54.6)	158 (70.9)	38 (47.5)	33 (52.4)	102 (93.6)	331 (69.7)
Missing	198 (34.9)	188 (32.2)	386 (33.5)	28 (35.0)	92 (19.4)	120 (21.6)	25 (37.9)	97 (35.5)	122 (36.0)	39 (17.5)	30 (37.5)	22 (34.9)	1 (0.9)	92 (19.4)
Site of primary tumour														
Other	284 (50.1)	257 (44.0)	541 (47.0)	32 (40.0)	245 (51.6)	277 (49.9)	26 (39.4)	106 (38.8)	132 (38.9)	130 (58.3)	40 (50.0)	17 (27.0)	58 (53.2)	245 (51.6)
Extremities	129 (22.8)	143 (24.5)	272 (23.6)	27 (33.8)	152 (32.0)	179 (32.3)	17 (25.8)	73 (26.7)	90 (26.5)	76 (34.1)	15 (18.8)	12 (19.0)	49 (45.0)	152 (32.0)
Missing	154 (27.2)	184 (31.5)	338 (29.4)	21 (26.3)	78 (16.4)	99 (17.8)	23 (34.8)	94 (34.4)	117 (34.5)	17 (7.6)	25 (31.3)	34 (54.0)	2 (1.8)	78 (16.4)
Histology														
Leiomyosarcoma	192 (33.9)	180 (30.8)	372 (32.3)	23 (28.8)	128 (26.9)	151 (27.2)	25 (37.9)	79 (28.9)	104 (30.7)	66 (29.6)	25 (31.3)	13 (20.6)	24 (22.0)	128 (26.9)
Synovial sarcoma	32 (5.6)	59 (10.1)	91 (7.9)	10 (12.5)	71 (14.9)	81 (14.6)	6 (9.1)	29 (10.6)	35 (10.3)	37 (16.6)	8 (10.0)	7 (11.1)	19 (17.4)	71 (14.9)
Other	315 (55.6)	317 (54.3)	632 (54.9)	44 (55.0)	266 (56.0)	310 (55.9)	35 (53.0)	151 (55.3)	186 (54.9)	119 (53.4)	42 (52.5)	40 (63.5)	65 (59.6)	266 (56.0)
Missing	28 (4.9)	28 (4.8)	56 (4.9)	3 (3.8)	10 (2.1)	13 (2.3)	0 (0.0)	14 (5.1)	14 (4.1)	1 (0.4)	5 (6.3)	3 (4.8)	1 (0.9)	10 (2.1)
Prior surgery														
No surgery	60 (10.6)	57 (9.8)	117 (10.2)	10 (12.5)	19 (4.0)	29 (5.2)	3 (4.5)	25 (9.2)	28 (8.3)	8 (3.6)	11 (13.8)	0 (0.0)	0 (0.0)	19 (4.0)
Non optimal surgery	104 (18.3)	77 (13.2)	181 (15.7)	18 (22.5)	23 (4.8)	41 (7.4)	10 (15.2)	64 (23.4)	74 (21.8)	11 (4.9)	12 (15.0)	0 (0.0)	0 (0.0)	23 (4.8)
Complete surgery	155 (27.3)	128 (21.9)	283 (24.6)	13 (16.3)	66 (13.9)	79 (14.2)	35 (53.0)	106 (38.8)	141 (41.6)	42 (18.8)	24 (30.0)	0 (0.0)	0 (0.0)	66 (13.9)
Unknown	248 (43.7)	322 (55.1)	570 (49.5)	39 (48.8)	367 (77.3)	406 (73.2)	18 (27.3)	78 (28.6)	96 (28.3)	162 (72.6)	33 (41.3)	63 (100.0)	109 (100.0)	367 (77.3)
Prior radiotherapy														
No	435 (76.7)	390 (66.8)	825 (71.7)	58 (72.5)	279 (58.7)	337 (60.7)	43 (65.2)	191 (70.0)	234 (69.0)	115 (51.6)	57 (71.3)	41 (65.1)	66 (60.6)	279 (58.7)
Yes	119 (21.0)	171 (29.3)	290 (25.2)	19 (23.8)	153 (32.2)	172 (31.0)	23 (34.8)	82 (30.0)	105 (31.0)	66 (29.6)	22 (27.5)	22 (34.9)	43 (39.4)	153 (32.2)
Missing	13 (2.3)	23 (3.9)	36 (3.1)	3 (3.8)	43 (9.1)	46 (8.3)	0 (0)	0 (0)	0 (0)	42 (18.8)	1 (1.3)	0 (0.0)	0 (0.0)	43 (9.1)
Primary site involved														
No	195 (34.4)	219 (37.5)	414 (36.0)	35 (43.8)	244 (51.4)	279 (50.3)	36 (54.5)	112 (41.0)	148 (43.7)	122 (54.7)	42 (52.5)	22 (34.9)	58 (53.2)	244 (51.4)
Yes	310 (54.7)	288 (49.3)	598 (52.0)	37 (46.3)	192 (40.4)	229 (41.3)	25 (37.9)	133 (48.7)	158 (46.6)	86 (38.6)	38 (47.5)	17 (27.0)	51 (46.8)	192 (40.4)
Missing	62 (10.9)	77 (13.2)	139 (12.1)	8 (10.0)	39 (8.2)	47 (8.5)	5 (7.6)	28 (10.3)	33 (9.7)	15 (6.7)	0 (0.0)	24 (38.1)	0 (0.0)	39 (8.2)
Metastatic site involved														
No	79 (13.9)	99 (17.0)	178 (15.5)	10 (12.5)	49 (10.3)	59 (10.6)	10 (15.2)	45 (16.5)	55 (16.2)	22 (9.9)	14 (17.5)	5 (7.9)	8 (7.3)	49 (10.3)
Yes	426 (75.1)	408 (69.9)	834 (72.5)	62 (77.5)	387 (81.5)	449 (80.9)	51 (77.3)	200 (73.3)	251 (74.0)	186 (83.4)	66 (82.5)	34 (54.0)	101 (92.7)	387 (81.5)
Missing	62 (10.9)	77 (13.2)	139 (12.1)	8 (10.0)	39 (8.2)	47 (8.5)	5 (7.6)	28 (10.3)	33 (9.7)	15 (6.7)	0 (0.0)	24 (38.1)	0 (0.0)	39 (8.2)

### Patients treated with at least 6 cycles of treatment

Considering the 748 patients with at least 6 cycles of treatment and without progression before or at the end of treatment, the median PFS from randomisation was 9.4 months (95% confidence interval: 8.9–9.9) and median PFS from end of treatment was 4.3 months (95% confidence interval: 3.8–4.7) (Additional file 1: Table S4 shows the PFS per treatment regimen). PFS for the different histologies was comparable and is provided in Additional file 1: Table S5.

Median OS from randomisation was 19.5 months (95% confidence interval: 18.2–21.3) and median OS from end of treatment was 14.5 months (95% confidence interval: 12.8–16.1) (Additional file 1: Table S6). The median OS according to histology were approximately the same and are provided in Additional file 1: Table S7.

### Patients treated with exactly 6 cycles of treatment

Because longer treatment duration could lead to bias, we also did the analysis for patients treated with exactly 6 cycles. For this analysis, 475 patients were included (85.6% of the total receiving 6 cycles). The median PFS from randomisation was 8.7 months (95% confidence interval: 8.2–9.1) and the median PFS from end of treatment was 4.2 months (95% confidence interval: 3.7–4.8) (Additional file 1: Table S8). A significant effect of treatment on PFS was found, patients receiving doxorubicin monotherapy had a worse PFS compared to patients receiving doxorubicin 75 mg/m^2^ combined with ifosfamide 10 g/m^2^ combination therapy [p = 0.021 and p = 0.036 respectively, as already reported by Judson et al*.* [3]]. In this analysis, no significant effect of histology on PFS was found (Additional file 1: Table S9).

Median OS from randomisation for these patients was 20.1 months (95% confidence interval: 18.3–22.3 months) and median OS from end of treatment was 15.7 months (95% confidence interval: 14.0–17.8). There was no statistically significant effect of treatment regimen or histology on OS (Additional file 1: Tables S10 and S11).

### Patients treated with less than 6 cycles and no progressive disease

The progression-free survival for patients treated with less than 6 cycles of doxorubicin-containing treatment regimens was 3.8 months (95% confidence interval 3.5–4.3 months) from randomisation (Additional file 1: Table S12). OS was 10.0 months (95% confidence interval 9.1–10.8 months) (Additional file 1: Table S14). As there can be a bias due to the number of cycles given, no formal statistical comparisons were done. The median progression-free survival and OS for the different treatment regimens are shown in Additional file 1: Tables S13 and S15 respectively, but did not differ.

## Discussion

In this study, we report the progression-free and OS of patients completing 6 cycles of doxorubicin based chemotherapy who did not progress before completion of this treatment. Knowledge of the PFS and OS of patients completing 6 cycles of doxorubicin without progressive disease is essential for planning maintenance studies with cytotoxic chemotherapy or tyrosine kinase inhibitors. It is also important to know what percentage of the total number of patients receiving systemic therapy is likely to be available for such trials.

The prognosis of patients with metastatic STS remains poor, with a median OS of 12.8–14.3 months respectively in a recently reported study of first-line doxorubicin versus doxorubicin/ifosfamide [3]. More recent studies show a median OS around 18 months [4–6]. Although the addition of olaratumab to doxorubicin seemed to be promising based on the phase II data, the confirmatory phase III trial, ANNOUNCE, did not show any benefit of the addition of olaratumab to doxorubicin (median overall survival 20.4 versus 19.7 months) [7, 8]. Now, one of the other strategies that could be explored to improve the OS of STS patients is the addition of maintenance therapy after first-line chemotherapy. This is a well-established concept in colorectal cancer, non-small cell lung cancer and ovarian cancer [20–22]. Progression after first-line treatment can result in a deterioration in performance status making it difficult or impossible to administer second-line treatment. Maintenance treatment is intended to improve OS by prolonging the progression-free survival after first-line treatment by direct continuation of chemotherapy. In STS, this is even more a problem, because doxorubicin is first-line treatment and has a maximum safe cumulative dose of 450 mg/m^2^ (6 cycles), although even at this dose there is evidence of cardiac damage in a significant percentage of patients. Administration of higher cumulative doses, e.g. 600 mg/m^2^ (8 cycles) as in the olaratumab study, is only possible with the co-administration of the cardioprotective agent cardioxane since the risk of cardiotoxicity at this dose without cardioprotection is in the region of 50%. An alternative to doxorubicin would be the use of liposomal doxorubicin, which does not have the cardiotoxic potential of doxorubicin [16]. When considering maintenance treatment, one needs to take into account the risks of this therapy and the loss in quality of life caused by the maintenance treatment. Drugs that have some proven utility against sarcomas and could be used in maintenance treatment include pazopanib and trabectedin, which are both well-tolerated [23–25]. Although the concept of maintenance treatment after doxorubicin is attractive, maintenance studies had trouble recruiting due to the temporary registration and availability of olaratumab in most of the western world. Probably, these trials will now recruit more easily, because olaratumab failed in the phase III trial. For designing future studies of maintenance therapy in STS, data on PFS and OS in this setting are essential.

It is important to realise that of all patients included in the database, only 43.7% received 6 cycles or more and only 83.7% of these did not progress before the end of treatment (36.6% of all patients). Patients treated with more than 6 cycles have a similar OS as patients receiving exactly 6 cycles of doxorubicin, but patients receiving less than 6 cycles without progressive disease at the end of treatment have a worse survival. Based on this database study we roughly estimate that only one third of all patients (all patients receiving 6 or more cycles and no progressive disease at end of treatment) will qualify for maintenance treatment.

The PFS of 8.7 months and the OS of 20.1 months from randomisation is much longer than the mean OS of patients included in first line studies. Of course, this is an expected difference because responding patients will have a better prognosis compared to patients not responding to chemotherapy. On the other hand, this improved survival should be accounted for when planning maintenance studies and single arm phase II studies.

One of the major limitations of this study is the long interval between the first included patient and the last included patient. Ifosfamide was already available in the early years of this study, but trabectedin, pazopanib and gemcitabine/docetaxel are new second or later line treatments prolonging PFS and/or OS [19, 23, 26]. These new second line treatments will cause bias when comparing older regimens like doxorubicin 50 mg/m^2^ combined with ifosfamide 5 g/m^2^ to newer regimens like doxorubicin 75 mg/m^2^ combined with ifosfamide 10 g/m^2^. The improved supportive care over the years will increase this bias somewhat further.

In this study, treatment regimen had only a significant effect on PFS, with doxorubicin 75 mg/m^2^ combined with ifosfamide 10 g/m^2^ having the best PFS. No significant effect on OS was found, but a trend towards an increase in OS was found for patients with doxorubicin/ifosfamide combination therapy, which is more or less comparable with our study on this regimen, showing only a very little improvement in OS compared with doxorubicin 75 mg/m^2^ monotherapy [3]. The increase in PFS without an increase in OS in this study could be the effect of sequentially using these agents compared to using them concurrently. For other tumours like colorectal cancer it has been shown that sequential treatment is comparable to concurrent treatment [27]. Second, as the study design selects for responding patients, the difference in OS between this study and the EORTC 62012 study could be caused by the increased response rate with doxorubicin/ifosfamide.

Importantly, this study shows no effect of histology on the outcome of patients, although the number of separately studied subtypes was small. This is in contrast to earlier studies, showing a better survival in for example synovial sarcoma [28]. These differences could be caused by the low number of included patients in this study, or by the exclusion of patients with progression during treatment, thereby selecting for responding patients.

## Conclusions

This is the first study reporting the progression-free survival and OS of patients completing 6 cycles of doxorubicin containing treatment without progressive disease before completion of treatment. These data are important for future study design and daily patient care as one of the ways forwards to improve survival in advanced STS could be maintenance treatment for the minority of patients whose disease is sensitive to chemotherapy.

## Supplementary information

**Additional file 1.** Additional Tables.

## Data Availability

The data used in this manuscript is available on request. The data is stored at EORTC. For conditions and procedures to assess the data: https://www.eortc.org/data-sharing/

## Abbreviations

GIST: Gastro-intestinal stromal tumour
IQR: Interquartile range
MFH: Malignant fibrous histiocytoma
OS: Overall survival
PFS: Progression free survival
STS: Soft tissue sarcoma
