# Supplementary material for: Survival of soft tissue sarcoma patients after completing six cycles of first-line anthracycline containing treatment: an EORTC-STBSG database study
Source: Clin Sarcoma Res. 2020 Sep 9;10:18. doi: 10.1186/s13569-020-00137-5 (PMC7488114; doi:10.1186/s13569-020-00137-5)

**Table S1** Included patients per study and regimen

|  | | | | | | | | | | | | | |
| --- | --- | --- | --- | --- | --- | --- | --- | --- | --- | --- | --- | --- | --- |
|  | **Protocol** | | | | | | | | | | | | **Total (N=2045)** |
|  | **62012 (N=433)** | **62061 (N=38)** | **62091 (N=41)** | **62801 (N=94)** | **62842 (N=194)** | **62851 (N=538)** | **62883 (N=111)** | **62901 (N=107)** | **62903 (N=309)** | **62941 (N=39)** | **62962 (N=41)** | **62971 (N=100)** |  |
|  | **N**  **(%)** | **N**  **(%)** | **N**  **(%)** | **N**  **(%)** | **N**  **(%)** | **N**  **(%)** | **N**  **(%)** | **N**  **(%)** | **N**  **(%)** | **N**  **(%)** | **N**  **(%)** | **N**  **(%)** | **N**  **(%)** |
| **Treatment** |  |  |  |  |  |  |  |  |  |  |  |  |  |
| **DOX 75** | 216 (49.9) | 38 (100.0) | 41 (100.0) | 94 (100.0) | 0 (0.0) | 272 (50.6) | 0 (0.0) | 107 (100.0) | 0 (0.0) | 39 (100.0) | 41 (100.0) | 100 (100.0) | 948 (46.4) |
| **DOX 50-IFO 5** | 0 (0.0) | 0 (0.0) | 0 (0.0) | 0 (0.0) | 194 (100.0) | 266 (49.4) | 0 (0.0) | 0 (0.0) | 154 (49.8) | 0 (0.0) | 0 (0.0) | 0 (0.0) | 614 (30.0) |
| **DOX 75-IFO 5** | 0 (0.0) | 0 (0.0) | 0 (0.0) | 0 (0.0) | 0 (0.0) | 0 (0.0) | 111 (100.0) | 0 (0.0) | 155 (50.2) | 0 (0.0) | 0 (0.0) | 0 (0.0) | 266 (13.0) |
| **DOX 75-IFO 10** | 217 (50.1) | 0 (0.0) | 0 (0.0) | 0 (0.0) | 0 (0.0) | 0 (0.0) | 0 (0.0) | 0 (0.0) | 0 (0.0) | 0 (0.0) | 0 (0.0) | 0 (0.0) | 217 (10.6) |

**Table S2** Distribution of number of cycles by study.

|  | **Study** | | | | | | | | | | | | |
| --- | --- | --- | --- | --- | --- | --- | --- | --- | --- | --- | --- | --- | --- |
| **Number of cycles** | **62012** | **62061** | **62091** | **62801** | **62842** | **62851** | **62883** | **62901** | **62903** | **62941** | **62962** | **62971** | **Total** |
| **1** | 34 | 6 | 5 | 6 | 10 | 59 | 10 | 12 | 26 | 3 | 4 | 4 | 179 |
| **2** | 94 | 7 | 9 | 20 | 33 | 107 | 18 | 22 | 62 | 6 | 10 | 28 | 416 |
| **3** | 30 | 0 | 1 | 15 | 20 | 56 | 15 | 13 | 36 | 5 | 4 | 13 | 208 |
| **4** | 32 | 2 | 3 | 7 | 23 | 63 | 18 | 12 | 28 | 4 | 6 | 15 | 213 |
| **5** | 17 | 1 | 0 | 14 | 17 | 40 | 13 | 5 | 21 | 2 | 0 | 5 | 135 |
| **6** | 225 | 22 | 23 | 8 | 25 | 70 | 31 | 16 | 81 | 6 | 14 | 34 | 555 |
| **7** | 1 | 0 | 0 | 10 | 12 | 48 | 5 | 21 | 26 | 13 | 2 | 1 | 139 |
| **8** | 0 | 0 | 0 | 7 | 29 | 46 | 1 | 6 | 14 | 0 | 1 | 0 | 104 |
| **9** | 0 | 0 | 0 | 2 | 4 | 23 | 0 | 0 | 5 | 0 | 0 | 0 | 34 |
| **10** | 0 | 0 | 0 | 3 | 13 | 14 | 0 | 0 | 8 | 0 | 0 | 0 | 38 |
| **11** | 0 | 0 | 0 | 1 | 0 | 5 | 0 | 0 | 2 | 0 | 0 | 0 | 8 |
| **12** | 0 | 0 | 0 | 0 | 4 | 1 | 0 | 0 | 0 | 0 | 0 | 0 | 5 |
| **13** | 0 | 0 | 0 | 1 | 1 | 1 | 0 | 0 | 0 | 0 | 0 | 0 | 3 |
| **14** | 0 | 0 | 0 | 0 | 0 | 3 | 0 | 0 | 0 | 0 | 0 | 0 | 3 |
| **15** | 0 | 0 | 0 | 0 | 2 | 1 | 0 | 0 | 0 | 0 | 0 | 0 | 3 |
| **16** | 0 | 0 | 0 | 0 | 0 | 1 | 0 | 0 | 0 | 0 | 0 | 0 | 1 |
| **17** | 0 | 0 | 0 | 0 | 1 | 0 | 0 | 0 | 0 | 0 | 0 | 0 | 1 |

**Table S3a** distribution of histological subtype and grade in patients treated with more than 6 cycles

| **More than 6 cycles** | | | |
| --- | --- | --- | --- |
|  | **Pts who progress before or at the end of treatment (N=66)** | **Pts who did not progress before or at the end of treatment (N=273)** | **Total (N=339)** |
|  | **N (%)** | **N (%)** | **N (%)** |
| **histological cell type** |  |  |  |
| **MFH** | 5 (7.6) | 39 (14.3) | 44 (13.0) |
| **Fibrosarcoma** | 5 (7.6) | 13 (4.8) | 18 (5.3) |
| **Liposarcoma** | 6 (9.1) | 25 (9.2) | 31 (9.1) |
| **Leiomyosarcoma** | 25 (37.9) | 79 (28.9) | 104 (30.7) |
| **Rhabdomyosarcoma** | 2 (3.0) | 4 (1.5) | 6 (1.8) |
| **Angiosarcoma** | 2 (3.0) | 10 (3.7) | 12 (3.5) |
| **Synovial sarcoma** | 6 (9.1) | 29 (10.6) | 35 (10.3) |
| **Neurogenic sarcoma** | 5 (7.6) | 19 (7.0) | 24 (7.1) |
| **Miscellaneous** | 6 (9.1) | 27 (9.9) | 33 (9.7) |
| **Unclassified** | 4 (6.1) | 14 (5.1) | 18 (5.3) |
| **Missing** | 0 (0.0) | 14 (5.1) | 14 (4.1) |
| **Histopathological grade** |  |  |  |
| **I** | 8 (12.1) | 24 (8.8) | 32 (9.4) |
| **II** | 15 (22.7) | 59 (21.6) | 74 (21.8) |
| **III** | 18 (27.3) | 93 (34.1) | 111 (32.7) |
| **Missing** | 25 (37.9) | 97 (35.5) | 122 (36.0) |

**Table S3b** distribution of histological subtype and grade in patients treated with exactly 6 cycles

| **Exactly 6 cycles** | | | |
| --- | --- | --- | --- |
|  | **Pts who progress before or at the end of treatment (N=80)** | **Pts who did not progress before or at the end of treatment (N=475)** | **Total (N=555)** |
|  | **N (%)** | **N (%)** | **N (%)** |
| **histological cell type** |  |  |  |
| **MFH** | 5 (6.3) | 35 (7.4) | 40 (7.2) |
| **Fibrosarcoma** | 4 (5.0) | 8 (1.7) | 12 (2.2) |
| **Liposarcoma** | 4 (5.0) | 65 (13.7) | 69 (12.4) |
| **Leiomyosarcoma** | 23 (28.8) | 128 (26.9) | 151 (27.2) |
| **Rhabdomyosarcoma** | 0 (0.0) | 10 (2.1) | 10 (1.8) |
| **Angiosarcoma** | 3 (3.8) | 22 (4.6) | 25 (4.5) |
| **Synovial sarcoma** | 10 (12.5) | 71 (14.9) | 81 (14.6) |
| **Neurogenic sarcoma** | 10 (12.5) | 13 (2.7) | 23 (4.1) |
| **Miscellaneous** | 13 (16.3) | 92 (19.4) | 105 (18.9) |
| **Unclassified** | 5 (6.3) | 21 (4.4) | 26 (4.7) |
| **Missing** | 3 (3.8) | 10 (2.1) | 13 (2.3) |
| **Histopathological grade** |  |  |  |
| **I** | 6 (7.5) | 52 (10.9) | 58 (10.5) |
| **II** | 16 (20.0) | 162 (34.1) | 178 (32.1) |
| **III** | 30 (37.5) | 169 (35.6) | 199 (35.9) |
| **Missing** | 28 (35.0) | 92 (19.4) | 120 (21.6) |

**Table S3c** distribution of histological subtype and grade in patients treated with less than 6 cycles and stopped for other reasons then progression

| **Less than 6 cycles** | | | |
| --- | --- | --- | --- |
|  | **Pts who progress before or at the end of treatment (N=567)** | **Pts who did not progress before or at the end of treatment (N=584)** | **Total (N=1151)** |
|  | **N (%)** | **N (%)** | **N (%)** |
| **histological cell type** |  |  |  |
| **MFH** | 58 (10.2) | 79 (13.5) | 137 (11.9) |
| **Fibrosarcoma** | 11 (1.9) | 22 (3.8) | 33 (2.9) |
| **Liposarcoma** | 47 (8.3) | 47 (8.0) | 94 (8.2) |
| **Leiomyosarcoma** | 192 (33.9) | 180 (30.8) | 372 (32.3) |
| **Rhabdomyosarcoma** | 16 (2.8) | 16 (2.7) | 32 (2.8) |
| **Angiosarcoma** | 23 (4.1) | 14 (2.4) | 37 (3.2) |
| **Synovial sarcoma** | 32 (5.6) | 59 (10.1) | 91 (7.9) |
| **Neurogenic sarcoma** | 18 (3.2) | 29 (5.0) | 47 (4.1) |
| **Miscellaneous** | 93 (16.4) | 80 (13.7) | 173 (15.0) |
| **Unclassified** | 49 (8.6) | 30 (5.1) | 79 (6.9) |
| **Missing** | 28 (4.9) | 28 (4.8) | 56 (4.9) |
| **Histopathological grade** |  |  |  |
| **I** | 38 (6.7) | 30 (5.1) | 68 (5.9) |
| **II** | 140 (24.7) | 162 (27.7) | 302 (26.2) |
| **III** | 191 (33.7) | 204 (34.9) | 395 (34.3) |
| **Missing** | 198 (34.9) | 188 (32.2) | 386 (33.5) |

**Table S3d** distribution of histological subtype and grade in patients treated with exactly 6 cycles according to treatment protocol

| **Exactly 6 cycles - no PD** | | | | | |
| --- | --- | --- | --- | --- | --- |
|  | **DOX 75 (N=223)** | **DOX 50-IFO 5 (N=80)** | **DOX 75-IFO 5 (N=63)** | **DOX 75-IFO 10 (N=109)** | **Total (N=475)** |
|  | **N (%)** | **N (%)** | **N (%)** | **N (%)** | **N (%)** |
| **histological cell type** |  |  |  |  |  |
| **MFH** | 9 (4.0) | 12 (15.0) | 7 (11.1) | 7 (6.4) | 35 (7.4) |
| **Fibrosarcoma** | 2 (0.9) | 1 (1.3) | 3 (4.8) | 2 (1.8) | 8 (1.7) |
| **Liposarcoma** | 36 (16.1) | 7 (8.8) | 6 (9.5) | 16 (14.7) | 65 (13.7) |
| **Leiomyosarcoma** | 66 (29.6) | 25 (31.3) | 13 (20.6) | 24 (22.0) | 128 (26.9) |
| **Rhabdomyosarcoma** | 6 (2.7) | 1 (1.3) | 2 (3.2) | 1 (0.9) | 10 (2.1) |
| **Angiosarcoma** | 12 (5.4) | 2 (2.5) | 2 (3.2) | 6 (5.5) | 22 (4.6) |
| **Synovial sarcoma** | 37 (16.6) | 8 (10.0) | 7 (11.1) | 19 (17.4) | 71 (14.9) |
| **Neurogenic sarcoma** | 4 (1.8) | 4 (5.0) | 5 (7.9) | 0 (0.0) | 13 (2.7) |
| **Miscellaneous** | 40 (17.9) | 11 (13.8) | 8 (12.7) | 33 (30.3) | 92 (19.4) |
| **Unclassified** | 10 (4.5) | 4 (5.0) | 7 (11.1) | 0 (0.0) | 21 (4.4) |
| **Missing** | 1 (0.4) | 5 (6.3) | 3 (4.8) | 1 (0.9) | 10 (2.1) |
| **Histopathological grade** |  |  |  |  |  |
| **I** | 26 (11.7) | 12 (15.0) | 8 (12.7) | 6 (5.5) | 52 (10.9) |
| **II** | 78 (35.0) | 12 (15.0) | 19 (30.2) | 53 (48.6) | 162 (34.1) |
| **III** | 80 (35.9) | 26 (32.5) | 14 (22.2) | 49 (45.0) | 169 (35.6) |
| **Missing** | 39 (17.5) | 30 (37.5) | 22 (34.9) | 1 (0.9) | 92 (19.4) |

**Table S4** Progression free survival of patients treated with >6 cycles

| **Treatment** | **Patients** | **Observed Events** | **Median (95% CI) (Months)** |
| --- | --- | --- | --- |
| **PFS from Randomisation** | | |  |
| DOX 75 | 336 | 308 | 8.48 (7.92, 9.10) |
| DOX 50-IFO 5 | 215 | 188 | 10.61 (9.82, 11.70) |
| DOX 75-IFO 5 | 88 | 81 | 9.31 (8.25, 11.60) |
| DOX 75-IFO 10 | 109 | 98 | 9.66 (8.77, 11.37) |
| Total | 748 | 675 | 9.40 (8.94, 9.89) |
| **PFS from End of treatment** | | |  |
| DOX 75 | 336 | 308 | 3.42 (3.12, 4.07) |
| DOX 50-IFO 5 | 215 | 188 | 4.70 (3.68, 5.68) |
| DOX 75-IFO 5 | 88 | 81 | 4.93 (3.61, 6.97) |
| DOX 75-IFO 10 | 109 | 98 | 4.99 (4.37, 6.67) |
| Total | 748 | 675 | 4.27 (3.84, 4.73) |

**Table S5** Progression free survival from End of treatment by histology for patients treated with >6 cycles

| **Histology** | **Patients (N)** | **Observed Events (O)** | **Median (95% CI) (Months)** |
| --- | --- | --- | --- |
| **DOX 75** | | |  |
| Leiomyosarcoma | 103 | 97 | 3.42 (2.92, 4.44) |
| Synovial sarcoma | 44 | 41 | 3.42 (2.07, 4.34) |
| Other | 183 | 166 | 3.42 (2.76, 4.44) |
| **DOX 50-IFO 5** | | |  |
| Leiomyosarcoma | 58 | 52 | 3.25 (2.10, 4.53) |
| Synovial sarcoma | 26 | 25 | 3.81 (2.14, 5.62) |
| Other | 120 | 101 | 6.93 (5.03, 8.44) |
| **DOX 75-IFO 5** | | |  |
| Leiomyosarcoma | 22 | 21 | 3.99 (2.60, 7.36) |
| Synovial sarcoma | 11 | 10 | 3.19 (0.92, 11.93) |
| Other | 50 | 45 | 6.34 (3.15, 10.09) |
| **DOX 75-IFO 10** | | |  |
| Leiomyosarcoma | 24 | 22 | 4.90 (2.92, 8.51) |
| Synovial sarcoma | 19 | 19 | 4.24 (2.96, 8.28) |
| Other | 65 | 56 | 5.13 (4.37, 7.43) |

**Table S6** Overall survival of patients treated with >6 cycles

| **Treatment** | **Patients (N)** | **Observed Events (O)** | **Median (95% CI) (Months)** |
| --- | --- | --- | --- |
| **OS from Randomisation** | | |  |
|  |  |  |  |
| DOX 75 | 336 | 237 | 18.73 (16.99, 21.88) |
| DOX 50-IFO 5 | 215 | 162 | 18.92 (16.66, 21.49) |
| DOX 75-IFO 5 | 88 | 77 | 19.19 (15.01, 23.75) |
| DOX 75-IFO 10 | 109 | 83 | 23.59 (19.32, 28.19) |
| Total | 748 | 559 | 19.48 (18.20, 21.29) |
| **OS from End of treatment** | | |  |
| DOX 75 | 336 | 237 | 13.96 (11.99, 16.76) |
| DOX 50-IFO 5 | 215 | 162 | 12.81 (10.94, 16.10) |
| DOX 75-IFO 5 | 88 | 77 | 15.05 (10.58, 18.89) |
| DOX 75-IFO 10 | 109 | 83 | 18.89 (14.95, 23.79) |
| Total | 748 | 559 | 14.52 (12.78, 16.10) |

**Table S7** Overall survival from End of treatment by histology for patients treated with >6 cycles

| **Histology** | **Patients (N)** | **Observed Events (O)** | **Median (95% CI) (Months)** | **Hazard Ratio (95% CI)** |
| --- | --- | --- | --- | --- |
| **DOX 75** | | |  |  |
| Leiomyosarcoma | 103 | 70 | 16.59 (11.17, 22.11) | 1.00 |
| Synovial sarcoma | 44 | 36 | 14.23 (9.30, 18.43) | 1.18 (0.79, 1.76) |
| Other | 183 | 127 | 12.94 (11.27, 16.76) | 1.08 (0.80, 1.44) |
| **DOX 50-IFO 5** | | |  | |
| Leiomyosarcoma | 58 | 49 | 10.68 (8.08, 13.08) | 1.00 |
| Synovial sarcoma | 26 | 22 | 12.29 (7.56, 16.10) | 1.10 (0.66, 1.83) |
| Other | 119 | 80 | 18.63 (13.96, 22.34) | 0.56 (0.39, 0.80) |
| **DOX 75-IFO 5** | | |  | |
| Leiomyosarcoma | 22 | 21 | 15.97 (9.20, 22.37) | 1.00 |
| Synovial sarcoma | 11 | 10 | 14.78 (4.73, 26.71) | 1.27 (0.60, 2.71) |
| Other | 50 | 42 | 11.53 (7.75, 20.47) | 0.93 (0.55, 1.58) |
| **DOX 75-IFO 10** | | |  | |
| Leiomyosarcoma | 24 | 20 | 17.35 (9.99, 26.71) | 1.00 |
| Synovial sarcoma | 19 | 17 | 18.89 (8.15, 25.10) | 1.37 (0.71, 2.63) |
| Other | 65 | 46 | 18.04 (11.37, 27.17) | 0.85 (0.50, 1.44) |

**Table S8** Progression free survival of patients treated with exactly 6 cycles

| **Treatment** | **Patients (N)** | **Observed Events (O)** | **Median (95% CI) (Months)** | **Hazard Ratio (95% CI)** | **P-Value (Score test)** |
| --- | --- | --- | --- | --- | --- |
| **PFS from Randomisation** | | |  |  |  |
| DOX 75 | 223 | 209 | 7.59 (7.23, 8.38) | 1.00 | 0.021 (df=3) |
| DOX 50-IFO 5 | 80 | 74 | 8.85 (7.33, 10.81) | 0.84 (0.65, 1.10) |  |
| DOX 75-IFO 5 | 63 | 59 | 9.10 (7.36, 11.40) | 0.74 (0.55, 0.99) |  |
| DOX 75-IFO 10 | 109 | 98 | 9.66 (8.77, 11.37) | 0.71 (0.56, 0.90) |  |
| Total | 475 | 440 | 8.67 (8.18, 9.13) |  |  |
| **PFS from End of treatment** | | |  |  |  |
| DOX 75 | 223 | 209 | 3.38 (2.73, 4.07) | 1.00 | 0.036 (df=3) |
| DOX 50-IFO 5 | 80 | 74 | 4.47 (3.06, 5.88) | 0.86 (0.66, 1.12) |  |
| DOX 75-IFO 5 | 63 | 59 | 4.73 (3.12, 6.97) | 0.75 (0.56, 1.00) |  |
| DOX 75-IFO 10 | 109 | 98 | 4.99 (4.37, 6.67) | 0.73 (0.57, 0.92) |  |
| Total | 475 | 440 | 4.24 (3.71, 4.80) |  |  |

**Table S9** PFS from End of treatment by histology for patients treated with exactly 6 cycles

| **histology** | **Patients (N)** | **Observed Events (O)** | **Median (95% CI) (Months)** |
| --- | --- | --- | --- |
| **DOX 75** | | |  |
| Leiomyosarcoma | 66 | 64 | 3.19 (2.60, 4.73) |
| Synovial sarcoma | 37 | 35 | 2.89 (1.94, 4.07) |
| Other | 119 | 110 | 3.71 (2.27, 5.09) |
| **DOX 50-IFO 5** | | |  |
| Leiomyosarcoma | 25 | 23 | 3.29 (2.04, 5.88) |
| Synovial sarcoma | 8 | 8 | 4.09 (0.03, 14.23) |
| Other | 42 | 38 | 7.43 (3.48, 9.63) |
| **DOX 75-IFO 5** | | |  |
| Leiomyosarcoma | 13 | 12 | 3.68 (2.37, 6.51) |
| Synovial sarcoma | 7 | 6 | 3.19 (0.92, 14.78) |
| Other | 40 | 38 | 5.80 (3.09, 10.09) |
| **DOX 75-IFO 10** | | |  |
| Leiomyosarcoma | 24 | 22 | 4.90 (2.92, 8.51) |
| Synovial sarcoma | 19 | 19 | 4.24 (2.96, 8.28) |
| Other | 65 | 56 | 5.13 (4.37, 7.43) |

**Table S10** Overall survival of patients treated with exactly 6 cycles

| **Treatment** | **Patients (N)** | **Observed Events (O)** | **Median (95% CI) (Months)** | **Hazard Ratio (95% CI)** | **P-Value (Score test)** |
| --- | --- | --- | --- | --- | --- |
| **OS from Randomisation** | | |  |  |  |
| DOX 75 | 223 | 148 | 18.96 (17.08, 22.34) | 1.00 | 0.340 (df=3) |
| DOX 50-IFO 5 | 80 | 63 | 20.11 (15.67, 24.61) | 1.08 (0.81, 1.46) |  |
| DOX 75-IFO 5 | 63 | 56 | 19.19 (15.01, 24.87) | 1.15 (0.84, 1.56) |  |
| DOX 75-IFO 10 | 109 | 83 | 23.59 (19.32, 28.19) | 0.86 (0.66, 1.12) |  |
| Total | 475 | 350 | 20.14 (18.30, 22.34) |  |  |
| **OS from End of treatment** | | |  |  |  |
| DOX 75 | 223 | 148 | 14.59 (12.55, 17.81) | 1.00 | 0.356 (df=3) |
| DOX 50-IFO 5 | 80 | 63 | 14.52 (11.53, 20.30) | 1.09 (0.81, 1.47) |  |
| DOX 75-IFO 5 | 63 | 56 | 15.05 (10.58, 20.47) | 1.15 (0.85, 1.57) |  |
| DOX 75-IFO 10 | 109 | 83 | 18.89 (14.95, 23.79) | 0.87 (0.66, 1.14) |  |
| Total | 475 | 350 | 15.74 (14.00, 17.81) |  |  |

**Table S11** Overall survival from End of treatment by histology for patients treated with exactly 6 cycles

| **histology** | **Patients (N)** | **Observed Events (O)** | **Median (95% CI) (Months)** |
| --- | --- | --- | --- |
| **DOX 75** | | |  |
| Leiomyosarcoma | 66 | 38 | 17.31 (12.55, 28.88) |
| Synovial sarcoma | 37 | 30 | 14.23 (9.30, 18.43) |
| Other | 119 | 80 | 14.00 (11.63, 18.27) |
| **DOX 50-IFO 5** | | |  |
| Leiomyosarcoma | 25 | 22 | 13.08 (8.64, 23.59) |
| Synovial sarcoma | 8 | 7 | 12.52 (7.56, 16.95) |
| Other | 42 | 29 | 20.76 (13.70, 30.62) |
| **DOX 75-IFO 5** | | |  |
| Leiomyosarcoma | 13 | 12 | 15.05 (11.33, 27.10) |
| Synovial sarcoma | 7 | 6 | 13.37 (2.50, 26.71) |
| Other | 40 | 35 | 15.31 (7.06, 21.85) |
| **DOX 75-IFO 10** | | |  |
| Leiomyosarcoma | 24 | 20 | 17.35 (9.99, 26.71) |
| Liposarcoma | 19 | 17 | 18.89 (8.15, 25.10) |
| Other | 65 | 46 | 18.04 (11.37, 27.17) |

**Table S12** Progression free survival of patients treated with less than 6 cycles *AND* no progressive disease before end of treatment

| **Treatment** | **Patients (N)** | **Observed Events (O)** | **Median (95% CI) (Months)** |
| --- | --- | --- | --- |
| **PFS from Randomisation** | | |  |
| DOX 75 | 233 | 222 | 2.76 (2.27, 3.09) |
| DOX 50-IFO 5 | 169 | 155 | 3.88 (3.32, 4.90) |
| DOX 75-IFO 5 | 111 | 107 | 6.93 (5.85, 8.11) |
| DOX 75-IFO 10 | 71 | 65 | 5.09 (3.84, 7.29) |
| Total | 584 | 549 | 3.81 (3.45, 4.30) |

**Table S13** PFS from End of treatment by histology for patients treated with less than 6 cycles *AND* no progressive disease before end of treatment

|  | | |  |
| --- | --- | --- | --- |
| **histology** | **Patients (N)** | **Observed Events (O)** | **Median (95% CI) (Months)** |
| **DOX 75** | | |  |
| Leiomyosarcoma | 53 | 52 | 3.12 (1.71, 3.88) |
| Synovial sarcoma | 23 | 21 | 2.79 (1.68, 14.92) |
| Other | 147 | 140 | 2.56 (2.23, 2.96) |
| **DOX 50-IFO 5** | | |  |
| Leiomyosarcoma | 58 | 55 | 3.48 (2.79, 4.90) |
| Synovial sarcoma | 23 | 23 | 4.57 (3.09, 9.07) |
| Other | 78 | 70 | 3.75 (2.76, 5.19) |
| **DOX 75-IFO 5** | | |  |
| Leiomyosarcoma | 43 | 43 | 7.13 (3.84, 8.51) |
| Synovial sarcoma | 7 | 7 | 8.57 (6.14, 12.75) |
| Other | 54 | 50 | 6.21 (5.16, 9.07) |
| **DOX 75-IFO 10** | | |  |
| Leiomyosarcoma | 26 | 26 | 5.06 (2.66, 7.23) |
| Synovial sarcoma | 6 | 6 | 9.53 (2.79, 37.49) |
| Other | 38 | 32 | 4.63 (3.22, 8.18) |

**Table S14** Overall survival of patients treated with less than 6 cycles *AND* no progressive disease before end of treatment

| **Treatment** | **Patients (N)** | **Observed Events (O)** | **Median (95% CI) (Months)** |
| --- | --- | --- | --- |
| **OS from Randomisation** | | |  |
| DOX 75 | 233 | 194 | 8.15 (7.29, 9.76) |
| DOX 50-IFO 5 | 169 | 136 | 10.02 (8.21, 12.06) |
| DOX 75-IFO 5 | 111 | 103 | 12.12 (9.92, 13.93) |
| DOX 75-IFO 10 | 71 | 55 | 11.70 (9.95, 14.78) |
| Total | 584 | 488 | 10.02 (9.07, 10.81) |

**Table S15** Overall survival from End of treatment by histology for patients treated with less than 6 cycles *AND* no progressive disease before end of treatment

| **histology** | **Patients (N)** | **Observed Events (O)** | **Median (95% CI) (Months)** |
| --- | --- | --- | --- |
| **DOX 75** | | |  |
| Leiomyosarcoma | 53 | 47 | 5.85 (3.38, 9.43) |
| Synovial sarcoma | 23 | 13 | 17.05 (10.55, 32.10) |
| Other | 147 | 125 | 5.26 (4.04, 6.80) |
| **DOX 50-IFO 5** | | |  |
| Leiomyosarcoma | 58 | 50 | 6.31 (4.47, 8.77) |
| Synovial sarcoma | 23 | 19 | 9.00 (4.73, 21.65) |
| Other | 78 | 61 | 6.60 (4.76, 10.28) |
| **DOX 75-IFO 5** | | |  |
| Leiomyosarcoma | 43 | 41 | 8.31 (5.98, 11.70) |
| Synovial sarcoma | 7 | 7 | 11.89 (7.92, 19.12) |
| Other | 54 | 48 | 9.99 (5.03, 13.90) |
| **DOX 75-IFO 10** | | |  |
| Leiomyosarcoma | 26 | 24 | 9.48 (7.56, 12.98) |
| Liposarcoma | 6 | 4 | 15.28 (6.31, N) |
| Other | 38 | 26 | 8.61 (5.68, 17.02) |

**Supplementary figure**

**Supplementary figure 1** Definition of end of treatment


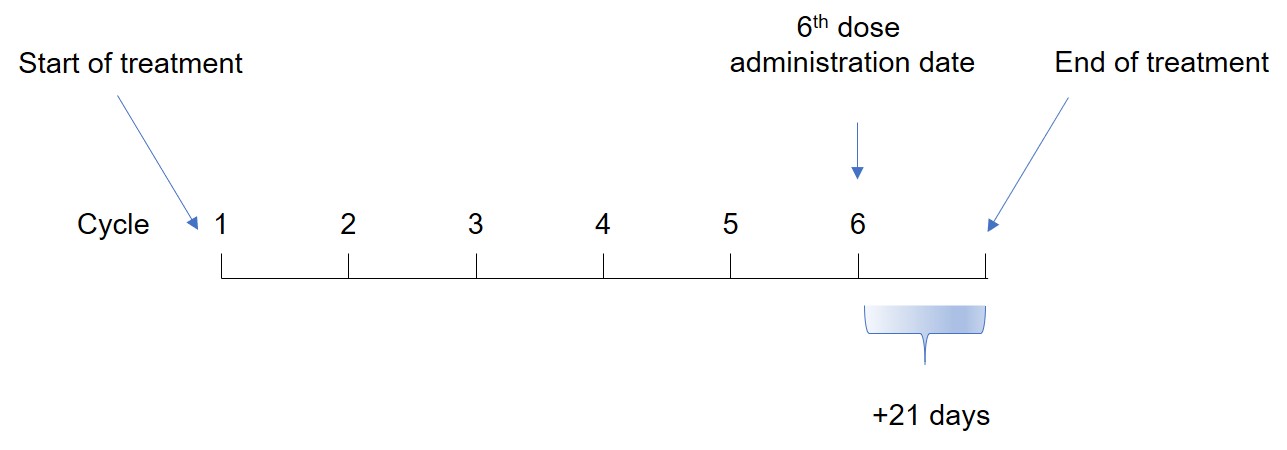

Supplement: Supplementary file 1 — Additional file 1. Additional Tables. [file 13569_2020_137_MOESM1_ESM.docx]
